# CD1d+ Goblet Cells Expand Colon-Resident Immature, Intermediate and Differentiated iNKT Cells to Limit Colitis

**DOI:** 10.21203/rs.3.rs-7603392/v1

**Published:** 2025-09-25

**Authors:** Vini John, Bibiana Barrios, Jacqueline D. Wang, Sreeram Udayan, Kathryn A Knoop, Alexandria N Floyd, Elisabeth L Joyce, Ellen M Schill, Sushma Sundas, Keely G. McDonald, Reyka G. Jayasinghe, Melissa Mavers, Richard S. Blumberg, Chyi Song Hsieh, Brigida Rusconi, Rodney D. Newberry

**Affiliations:** 1 The Saban Research Institute, Children’s Hospital Los Angeles, Los Angeles, CA, USA; 2 Division of Gastroenterology, Department of Internal Medicine, Washington University in Saint Louis School of Medicine, St. Louis, MO, USA 63110; 3 Division of Gastroenterology, Hepatology & Nutrition, Department of Pediatrics, Washington University in Saint Louis School of Medicine, St. Louis, MO, USA 63110; 3 School of Biochemistry and Immunology, Trinity College Dublin, Dublin, Ireland; 4 Department of Immunology, Mayo Clinic, Rochester, MN 55901; 5 Divsion of Newborn Medicine, Department of Pediatrics, Washington University in Saint Louis School of Medicine, St. Louis, MO, USA 63110; 6 Division of Oncology, Department of Medicine, Washington University in Saint Louis School of Medicine, St. Louis, MO, USA 63110; 7 Division of Hematology and Oncology, Department of Pediatrics, Washington University in Saint Louis School of Medicine, St. Louis, MO, USA 63110; 8 Division of Gastroenterology, Department of Medicine, Brigham and Women’s Hospital, Harvard Medical School, Boston, Massachusetts 02115; 9 Division of Rheumatology, Department of Internal Medicine, Washington University in Saint Louis School of Medicine, St. Louis, MO, USA 63110

## Abstract

Invariant Natural Killer T (iNKT) cells recognize glycolipid antigens presented on CD1d and rapidily respond to direct immune responses. iNKT cells develop in the thymus and migrate to peripheral tissues in what has been presumed to be differentiated/committed states. Accordingly, colonic iNKT cells are established in early life, considered to remain ‘fixed’ post-weaning, and determine life-long colitis susceptibility. Using single cell RNA sequencing (scRNA-seq), we demonstrate that humans and mice contain colonic iNKT populations transcriptionally resembling immature and intermediate thymic iNKT precursors. Contrary to prevailing paradigms, we demonstrate colonic iNKT cell populations expand in adult mice when CD1d expressing colonic goblet cells form goblet cell-associated antigen passages. Expansion preferentially affected intermediate iNKT cells, was durable, and protective in a colitis model. These studies reveal that the adult colon harbors iNKT cells with retained plasticity and uncover a novel role for goblet cells as unconventional antigen presenting cells regulating this axis.

## Introduction

The mammalian intestinal immune system is dynamic, continually responding to the environment to promote tolerance to luminal antigens in the homeostatic state while remaining poised to protect against potential pathogens during infection^1, 2, 3^. A contributor to this versatility and rapid response are gut invariant natural killer T (iNKT) cells, which display characteristics of both innate and adaptive immunity and are early responders to environmental stimuli^4, 5, 6, 7, 8^. iNKT cells express semi-invariant T cell receptors (TCR)^8, 9^, with mice expressing a TCR consisting of a Vα14Jα18 containing α chain paired with either Vβ2, Vβ7, Vβ8.1, Vβ8.2 or Vβ8.3 containing β chain and humans expressing a Vα24Jα18 α chain largely paired with Vβ11^10, 11^. iNKT cells have specificity for environmental, including microbial, and self-glycolipid ligands presented by a non-polymorphic MHC Class I like molecule, CD1d^12^. Despite being present in low numbers, iNKT cells hold significant importance in immune responses. Upon stimulation by glycolipids presented on CD1d, iNKT cells rapidly release a variety of cytokines, chemokines, and mediators with the capacity to recruit or activate components of the innate and adaptive immune system including dendritic cells (DCs), neutrophils, macrophages, NK cells, B cells, and conventional T cells^13, 14, 15, 16, 17, 18, 19^.

In contrast to ‘conventional’ TCRαβ T cells, which bear an almost limitless TCR repertoire, are selected by classical MHC molecules in the thymus, and, with the exception of thymically derived ‘natural’ T regulatory (Treg) cells, migrate to the periphery in an undifferentiated state, iNKT cells develop in the thymus and prevailing concepts suggest that they migrate to the periphery in a largely differentiated or ‘committed’ states^20^. Recent studies have classified iNKT cells into subsets that bear similarities to helper T cells and innate lymphoid cells (ILCs) based upon transcription factors required for their development and the cytokines produced^21,22, 23^. PLZF (promyelocytic leukemia zinc finger protein; *zbtb16*) is expressed by precursor thymocytes bearing the invariant iNKT TCR that have become committed to the iNKT cell lineage. While required for iNKT cell development, iNKT cell subsets express varying levels of PLZF, with iNKT1 cells subset exhibiting low PLZF expression, iNKT17 cells having intermediate PLZF expression, and iNKT2 cells showing the highest PLZF expression^24^. iNKT1 cells are also characterized by the expression of T-bet and the production of IFN-γ, while iNKT2 cells are characterized by GATA3 expression and IL-4 and IL-13 production, and iNKT17 cells are characterized by Rorγt expression and IL-17 production. Beyond these three subsets, Bcl-6 dependent follicular iNKT cells (iNKT_FH_) that promote antibody affinity maturation^10, 25^ and IL-10 producing iNKT cells (iNKT10), which are enriched in adipose tissue have also been identified^26, 27^. Interestingly, iNKT10 cells appear to express little PLZF and IL-10 production was found to be dependent upon E4BP4^28^. The processes controlling iNKT subset fate/decision within the thymus are not well understood, but in part are due to TCR signal strength, which is largely determined by the avidity for glycolipid antigens presented on CD1d as a function of the level of TCR expression and TCR affinity within an individual iNKT cell^29^.

While variations in iNKT cell populations across peripheral tissues is well accepted, the mechanisms that establish and regulate peripheral iNKT cells are incompletely defined. Functionally iNKT cells can exert pathologic or protective effects depending on the organ and disease setting, which likely relates to differences in the iNKT cell subsets involved^30^. Gut iNKT cells have been implicated in inflammatory bowel disease (IBD) pathogenesis and exacerbating intestinal inflammation in mouse models^31, 32, 33^, effects that can be partially mitigated by CD1d - dependent induction of IL-10 expression in intestinal epithelial cells^34^. Among gut iNKT cells, those residing in the colon have a particularly intriguing biology. Colonic iNKT cells are established during a defined preweaning period that is in part controlled by the gut microbiota, which can supply inhibitory sphingolipids to limit the colonic iNKT cell accumulation and by suppressing embryonically derived macrophages which otherwise promote the colonic residence of iNKT cells^31, 35, 36^. Accordingly, germ-free mice have an increased colonic iNKT cell numbers and have increased colitis susceptibility, which can be reversed by colonization with conventional gut microbiota before weaning, but not in later life^31^. Thus, early life events can fix a setpoint for colonic iNKT cells that determines lifelong colitis susceptibility.

In contrast to the above observations, emerging evidence challenges the concept that iNKT cells are terminally differentiated or committed upon thymic egress. CCR7 expressing iNKT cells can migrate to peripheral organs in an immature state and then complete differentiation in peripheral sites^37^. Further adding to the potential plasticity and complexity of peripheral iNKT cell subsets, responses to TGFβ differ between thymic, liver, and splenic iNKT cells^38^, and studies in mice revealed sex-specific transcriptional profiles in iNKT cells responding to glycolipid antigens^39, 40, 41^. Thus, while in comparison to traditional T cells, iNKT cells largely leave the thymus in more differentiated, or committed states, iNKT cells residing at peripheral sites may have more diversity and plasticity than previously thought, the extent of which is not well understood.

To better understand the diversity and functions of colonic iNKT cells, we performed single cell RNA sequencing (scRNA-seq) on colonic iNKT cells isolated from humans and mice. In addition to populations expressing the transcriptomic signatures characteristic of differentiated iNKT cell subsets, we unexpectedly identified colon iNKT cell populations with transcriptomic signatures resembling immature, intermediate, and cycling thymic iNKT cell states, raising the possibility of plasticity in colonic iNKT cells. Consistent with prior studies, luminal stimulatory glycolipid antigens did not expand the colonic iNKT cell population, however the colonic iNKT cell population expanded when colonic goblet cells formed goblet cell associated antigen passages (GAPs) and expansion was dependent upon goblet cell expression of CD1d. Colonic iNKT cell expansion was durable, preferentially affected an intermediately differentiated colonic iNKT cell population and was protective in a mouse colitis model. Thus, in contrast to prior concepts, we find that colonic iNKT cells contain incompletely differentiated/developed populations, and that these cells are manipulable post-weaning. Further, these findings uncover a new role for intestinal goblet cells as unconventional antigen presenting cells capable of acquiring and delivering luminal glycolipid antigens to stimulate colonic iNKT cells.

## Results

### Human and Mouse colonic iNKT cells exhibit transcriptomic signatures spanning a range of maturation states

**Extended Data Fig.1:**
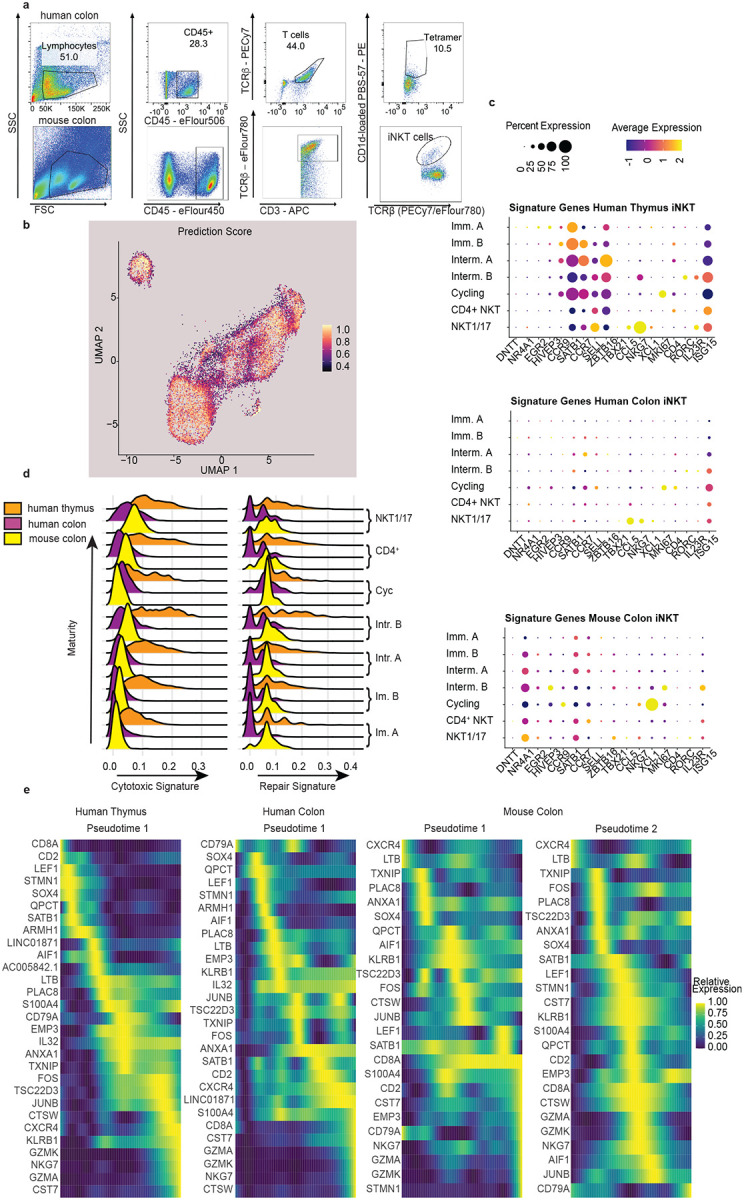
Human colonic iNKT cell subsets have transcriptomic signatures that are closely related to human thymic iNKT cell subsets and have limited repair and cytotoxic signatures. **a** Gating strategy for sorted human and mouse colonic iNKT cells gated on viable cells. **b** Prediction score for annotation transfer of human thymic iNKT cell subsets to human colonic iNKT cell subsets. **c** Expression of canonical genes for human thymic iNKT cell and MAIT cell subsets as identified by Bugaut *et al*. in human thymic, human colon, and mouse colon iNKT cell subsets. **d** Human thymic, human colon, and mouse colon iNKT cell subsets module score of cytotoxic and repair signatures based on previously published gene lists^46^. **e** Pseudotime of human thymic, human colon, and mouse colon iNKT cell subsets based on the top 30 temporally expressed genes in human thymic iNKT cell subsets^43^.

**Fig. 1:**
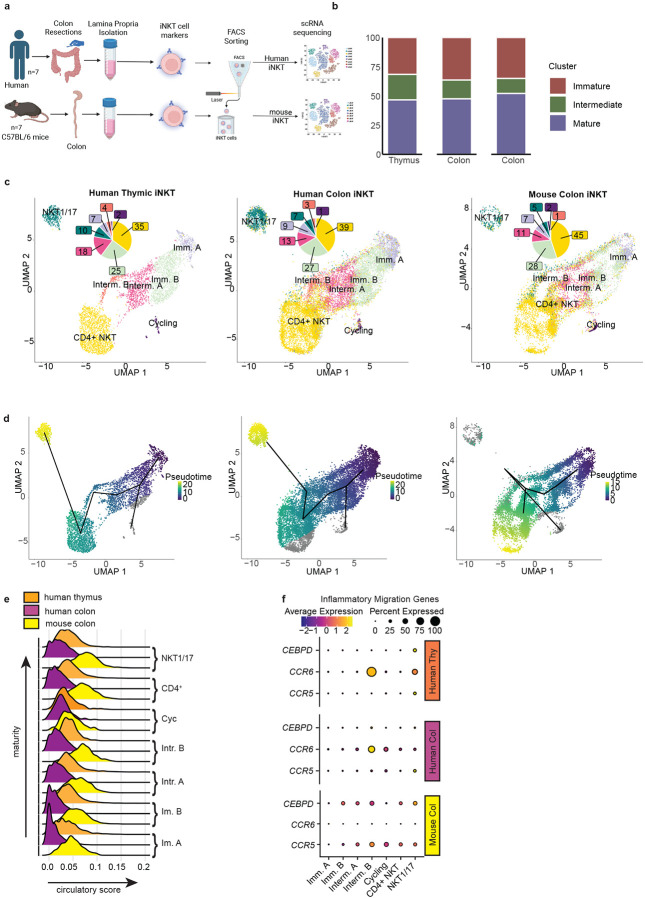
Human and mouse colonic iNKT cell populations contain immature, intermediate, and mature subsets. **a** Schematic of human and mouse colonic iNKT cell isolation, scRNA-seq, and analysis. **b** Barplots of the relative abundance of immature, intermediate, and mature iNKT subsets and **c** clustering and annotations of iNKT cells mapped using previously published thymic iNKT dataset and annotations^43^. **d** Trajectory analysis of human thymic and colon and mouse colon iNKT cells colored by pseudotime. **e** Expression of previously identified circulatory gene signatures in human thymus and colon and mouse colon iNKT cell subsets grouped by maturity. **f** Expression of previously identified inflammatory migration genes in human thymus and colon and mouse colon iNKT cell subsets.

Studies employing bulk and scRNA-seq of peripheral and thymic iNKT cells and MAIT cells in mice have identified multiple cellular subsets with overlapping transcriptional profiles. These shared transcriptional signatures have provided insights into iNKT cell and MAIT cell development within the thymus and better defined precursor cell stages^42^. In contrast more limited knowledge is available on the transcriptional profiles of human peripheral iNKTs cell subsets, with no studies evaluating the transcriptional profile of colonic iNKT cell populations using scRNAseq. To better understand the diversity and composition of the colonic iNKT cell populations, we performed scRNA-seq on flow cytometric cell sorted iNKT cells (CD45^+^, CD3^+^, TCRβ^+^, CD1d-loaded tetramer^+^) isolated from the colonic lamina propria (LP) of seven human colonic resection specimens (Schematic Fig. 1a, gating strategy Extended Data Fig. 1a). The resection specimens were surgical waste obtained from uninvolved macroscopically normal portions of colon from individuals undergoing surgery for colon polyps, diverticulitis, a small bowel tumor, and small bowel Crohn’s disease (detailed in Extended Data Table 1). A total of 13,909 human colonic iNKT cells passed initial quality control.

A recent cross-species study, including humans, analyzed thymic MAIT and iNKT cells and identified shared transcriptomic signatures for multiple mature, immature, and intermediate subsets/stages and their shared developmental trajectories^43^. To integrate our findings with the current understanding of iNKT cell subsets, we used this thymic iNKT cell dataset to analyze the human colonic iNKT cells transcriptomic signatures in our study. Using this dataset as an ‘anchor’ to identify known and novel iNKT cell populations we observed a high similarity between the human thymic iNKT cell populations, from the previously published data set, and the human colon iNKT cell populations, using the dataset we generated, including the presence and proportions of iNKT cells with immature, intermediate, and cycling transcriptomic signatures in the colon (Fig. 1b and Fig. 1c left and middle panels). To validate the clustering of the colonic iNKT cells based upon anchoring to the thymic iNKT cell dataset, we used a prediction score based upon gene signatures defining the clusters of human thymic iNKT cell subsets. We observed that the colonic iNKT cell clusters displayed a strong transcriptional correlation with the thymic iNKT cell subsets/stages including the immature, intermediate, cycling, and mature populations, with cycling, immature A, immature B, and mature populations displaying the strongest predictive score (Extended Data Fig. 1b). Moreover, canonical markers identified for the thymic iNKT cell subsets by Bugaut *et. al*.^43^ (Extended Data Fig. 1c upper panel) were similarly expressed by the corresponding colonic iNKT subsets, although to lesser degrees (Extended Data Fig. 1c middle panel).

The presence of immature and intermediate iNKT cell populations in the human colon was somewhat unexpected, as these populations are suggested to reside in the thymus and be precursors to mature populations which migrate to the periphery. Moreover, mouse studies have shown that colonic iNKT cells are established in early life under the control of the developing gut microbiota^31, 35, 36^, and colonic iNKT cells have not been thought to be manipulable in later life. Therefore, to determine if the presence of immature and intermediate iNKT cell populations extended to mice, we performed scRNA-seq on sorted colonic mouse iNKTs cells (CD45^+^, CD3^+^, TCRβ^+^, CD1d-loaded tetramer^+^) from 7 female C56BL/B6 mice (Schematic Fig. 1a), with 1107 colonic iNKT cells passing quality control. To allow for a cross-species comparison we defined orthologs and mapped the mouse colonic iNKTs on the same human thymic anchoring set used to map the human colonic iNKT cells. Like the human colon, we saw that the mouse colon contained populations with transcriptomic signature of all the iNKT subsets/stages identified in the human thymus with a similar distribution of immature, intermediate, and mature populations (Fig. 1b–c). The expression of canonical markers in the mouse colonic iNKT cell populations, as identified in thymic iNKT cell subsets/stages by Bugaut *et al*.^43^, was more varied when compared with the human colonic iNKT cell populations (Extended Data Fig. 1c lower panel), likely in part due to the cross species comparison.

To confirm the relationships of the immature, intermediate, and mature colonic iNKT populations to each other, we performed a pseudotime trajectory analysis. Human colonic iNKT populations showed a similar progression from immature to intermediate populations to mature clusters designated CD4^+^ and NKT1/17 based upon recently described transcriptional signatures 43 (Fig. 1d left and middle panels and Fig. Extended Data 1e). In mice the relationship between the immature, intermediate colonic iNKT populations was similar to the human thymus and colon. However, the relationship between the CD4^+^ and NKT1/17 cell subsets differed in the mouse colon resulting in a split trajectory terminating on iNKT CD4^+^ cell populations on one side and the NKT1/17 populations connecting with the cycling population on the other (Fig. 1d right panel, Fig. Extended Data 1e). This divergence in trajectories likely reflects both species differences and limitations of integrating cross species comparisons.

The unexpected prevalence of immature and intermediate iNKT populations in the colon raised the possibility that these might derive from thymic migratory populations as opposed to resident populations. To address this, we calculated the circulation score for each cell based on a previously published circulating gene signature associated with T cell recirculation^44^. In comparison to thymic MAIT cells which have a minimum circulatory score of 0.21 and a maximum score of 0.78, the circulatory signature was low in all iNKT cell subsets examined, with slight elevation in the intermediate B and more terminally differentiated NKT1/17 and CD4^+^ mouse colonic iNKT cell subsets (Fig. 1e). This slight increase in mouse but not human colonic iNKT cell populations could be related to the circulatory signature being defined using the mouse transcriptome and subsequent limitation of correlating this signature to human gene orthologues^44^. Despite this limitation, these findings are consistent with colonic iNKT cells in mice and humans largely being resident populations. Interestingly, in mouse MAIT cells the circulatory signature was only seen in thymus and lymph nodes, but not in the intestine^43^. The thymic iNKTs cell transcriptomes evaluated here were generated from the same study identifying the thymic MAIT transcriptomes and the circulating signature, suggesting there is a difference in circulation of MAIT and iNKT cells.

We further assessed expression of CEBPD, CCR5, and CCR6, which have been inferred to allow thymic CD4^+^ iNKT and iNKT 1/17 subsets to cross the endothelium and enter inflamed tissues^43, 45^. In contrast to thymic iNKT1/17 where a small proportion of cells highly express *CEBPD*, *CEBPD* expression was low in a small population of mouse and human colonic iNKTs (Fig. 1e). Human colonic and thymic intermediate B iNKT cells expressed *CCR6*, whereas *CCR6* expression was low in all mouse colonic iNKT cell populations (Fig. 1e). In contrast *CCR5* expression was greater in multiple mouse colonic iNKT populations (Fig. 1e).

We assessed functional specialization using previously defined repair and cytotoxic signatures^43, 46^. Consistent with prior studies, the cytotoxic signature in the human thymic iNKT cells was found in the CD4^+^ and iNKT1/17 clusters and to a lesser extent in more immature populations. The human colonic iNKT population also showed a cytotoxic signature that was strongest in the more differentiated subsets, but in general less than their human thymic counterparts (Extended Data Fig. 1d). Most cells in the mouse colonic iNKT cell populations displayed a slightly higher cytotoxicity signature when compared to human colonic iNKT cell populations, however this signature was markedly lower than that seen in the thymic iNKT cell populations (Extended Data Fig. 1d). Similar to the cytotoxic signatures, repair signatures appeared stronger in the more differentiated subsets in the human thymus (Extended Data Fig. 1d). The human colonic iNKT cell populations displayed a bimodal distribution of repair signature gene expression through almost all stages of maturation and interestingly mouse colonic iNKT cell populations had some of the stronger repair signatures when compared to both human thymic and colonic iNKT populations (Extended Data Fig. 1d). These observations suggest that the colonic iNKT cell populations and subsets may have less cytotoxic properties when compared with the thymus and may be tuned for tissue repair.

**Extended Data Fig.2:**
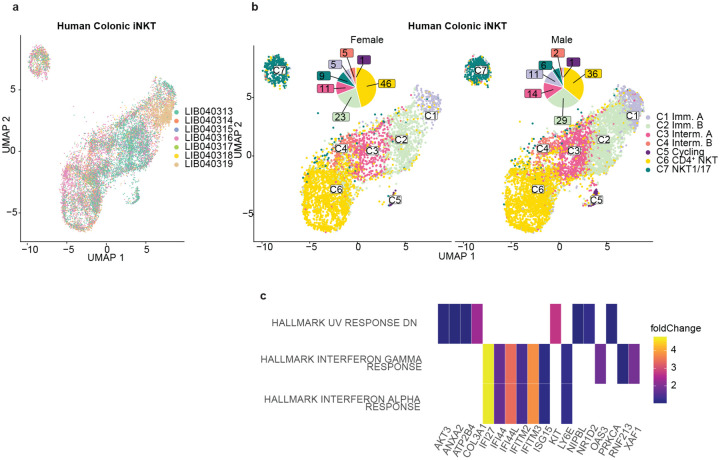
Human colonic iNKT have sex-specific transcriptional profiles at steady state. **a** Sorted iNKT cells from 7 donors are distributed throughout the iNKT cell clusters. **b** Clusters of human colonic iNKT cells separated by sex showed increased prevalence of Immature A and Intermediate B in male samples. **c** Pathway analysis of differentially expressed genes based on sex show increased responses to interferon gamma and alpha in human colonic iNKT from female subjects. **d** Table with demographic, surgery diagnosis, and medication for human subjects in this study. No patients were receiving antibiotics prior to surgery.

Finally, we assessed sex-based differences in human colonic iNKT cell populations. Sex based differences in iNKT cell populations have been documented in mice^39^, but have not been explored in humans. All human donors, regardless of sex, were represented in the identified clusters of colonic iNKT cells (Extended Data Table 1). However, the colonic iNKT cells from male donors were more skewed towards the immature and intermediate populations, and accordingly away from the mature populations (Extended Data Fig. 2b). Pathway analysis of differentially expressed genes based on sex in human colonic iNKT cells identified an increase in interferonγ and interferonα responses in female donors at baseline (Extended Data Fig. 2c), a finding similar to observations in mice^39^. Thus, human colonic iNKT cells recapitulate the sex-based transcriptional differences seen in mice.

### Luminal α-galactosylceramide (α-GalCer) expands small intestinal but not colonic iNKT cells.

**Fig. 2:**
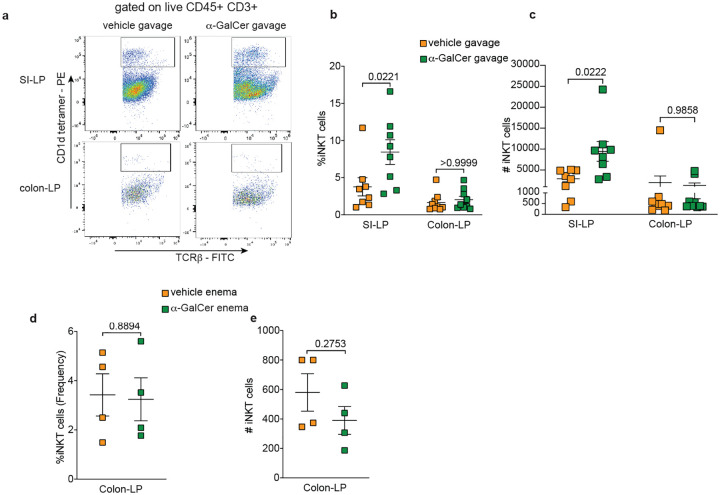
Lamina propria (LP) iNKT cells are expanded in the SI, but not colon, by exogenous luminal α-galactosyl ceramide (α-GalCer). **a** Representative FACS plots and graphical representation of **b** percentage and **c** numbers of iNKT cells (tetramer+ CD45^+^CD3^+^TCRβ^+^) in the SI and colon LP of vehicle and α-GalCer gavaged mice. **d** Percentage and **e** absolute numbers of iNKT cells in the colon LP of mice given vehicle and α-GalCer by enema. Each data point represents an individual mouse. Data presented here as mean SD and analyzed by a two-tailed student’s t test.

Current understanding is that iNKT cells seed the colon during a preweaning interval and their expansion/infiltration is terminated by the gut microbiota around the time of weaning, fixing the size, and relatedly the phenotype, of the colonic iNKT cell population for life^31^. This fixed population of colonic iNKT cells in part defines a threshold for iNKT cell mediated colonic diseases throughout life^31, 36^. Mechanistic support of this concept arises from observations that embryonic macrophages, which are suppressed by the gut microbiota around the time of weaning, support the establishment of the colonic iNKT cell population in early life^36^ and bacterial taxa present in the gut microbiota can produce inhibitory glycolipid antigens (sphingolipids) suppressing iNKT cell proliferation/expansion^35^. Our above observation that the adult colon contains immature and intermediate iNKT cell populations is potentially in conflict with the concept of a colonic iNKT cell population that is ‘fixed’. We therefore asked if supplying an exogenous stimulatory glycolipid antigen (α-galactosylceramide; α-GalCer) in the gut lumen could expand the gut iNKT cell population. Gavage of α-GalCer significantly increased the frequency and number of iNKT cells in the small intestine (SI) but had no effect on the iNKT cell population in the colon (Fig. 2a–c). The ability of gavaged α-GalCer to expand the SI iNKT population but not colonic iNKT cell population could be related to gavaged α-GalCer not reaching the colon. However, administering α-GalCer via enema was likewise ineffective at increasing the frequency or numbers of colonic iNKT cells (Fig. 2d–e), which would be consistent with the concept that the colonic iNKT cell population is not manipulable in later life.

### Colonic iNKT cells expand when colonic goblet cells form goblet cell-associated antigen passages

**Fig. 3:**
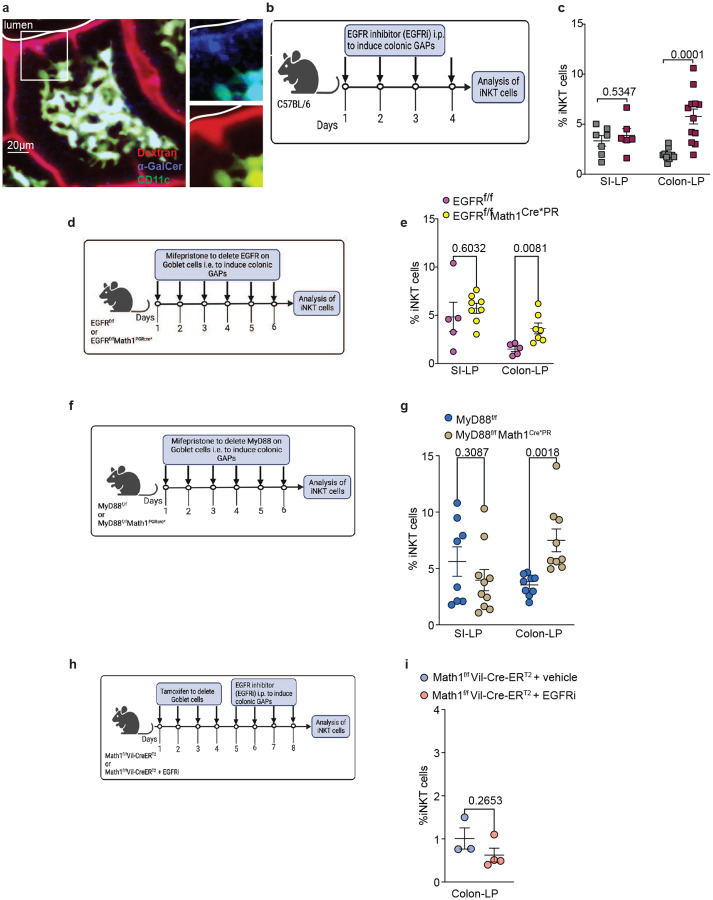
Colonic iNKT cells expand when GAPs are induced in the colon. **a** Photomicrograph from 2-Photon *in vivo* imaging of the ileum of a CD11c^YFP^ (green) reporter mouse given luminal α-GalCer (blue) and 10kD dextran (red). Images on the right are magnifications of inset in the left image showing (upper image) α-GalCer and CD11c^YFP^ and (lower image) dextran and CD11c^YFP^ **b** Schematic of EGFRi treatment to induce GAP formation. **c** Percentage of iNKT cells (tetramer^+^ of CD45^+^CD3^+^TCRβ^+^) in the SI-LP and Colon-LP of control and EGFRi treated mice. **d** Schematic of treatment and **e** Frequency of iNKT cells in the SI-LP and colon-LP of EGFR^f/f^ and EGFR^f/f^ Math1^PGRCre*^ mice. **f** Schematic of treatment and **g** frequency of SI-LP and colon-LP iNKT cells in MyD88^f/f^ and MyD88^f/f^ Math1^PGRCre*^ mice. **h** Schematic of tamoxifen and EGFRi treatment and **i** frequency of iNKT cells in the Colon-LP of Math1^f/f^ and Math1^f/f^Vil-Cre-ER^T2^ mice. Each data point represents one mouse. Data presented here as mean SD and analyzed by a two-tailed student’s t test.

To understand how α-GalCer was crossing the SI epithelium to be encountered by iNKT cells, we performed two-photon imaging in anesthetized mice^47^. Luminally administered fluorescently labeled α-GalCer and fluorescent 10kD dextran were observed crossing the epithelium in columnar structures characteristic of goblet cell associated antigen passages (GAPs)^47^ (Fig. 3a). GAP formation is an atypical fluid phase endocytic process delivering luminal cargo to the transcytotic pathway^48^, providing luminal antigens to generate antigen specific T cell responses and playing an important role in induction and maintenance of tolerance to dietary and gut microbial antigens^49, 50^. Consistent with GAPs delivering α-GalCer and expanding the SI iNKT population, but not the colonic iNKT population, GAPs are present in the adult SI but largely absent in the colon in the homeostatic state due to goblet cell sensing of the gut microbiota, which transactivates the epidermal growth factor receptor (EGFR) and suppresses the ability of goblet cells to respond to acetylcholine (ACh) via the muscarinic acetylcholine receptor 4 (mAChR4) to form a GAPs^2, 47, 48^.

To investigate if colonic GAPs could deliver glycolipid antigens to expand colonic iNKT cells, we used pharmacologic and genetic approaches to override colonic GAP inhibition. Pharmacologic inhibition of EGFR activation (EGFRi; Fig. 3b–c), deletion of the EGFR in goblet cells (EGFR^f/f^Math1^PR*Cre^ mice; Fig. 3d–e), or deletion of MyD88 in goblet cells (MyD88^f/f^Math1^PR*Cre^ mice; Fig. 3f–g), which have been demonstrated to induce GAP formation in the colon^2, 51^, resulted in a significant increase in the colonic iNKT cell population, but not in the SI iNKT cell population, consistent with colonic GAPs delivering glycolipid antigens. The inability of these interventions to alter the SI iNKT cell population likely reflects that SI GAPs are present and not inhibited by microbial sensing or EGFR activation at steady state^2^, and therefore interventions such as EGFR inhibition or deletion of Myd88 in goblet cells has limited effects on SI GAPs. To confirm the effect of EGFRi on the expansion of iNKT cells was goblet cell dependent, we administered EGFRi to mice in which goblet cells were deleted (Math1^f/f^Vil-Cre-ER^T2^ mice). EGFRi did not induce colonic iNKT cell expansion in the absence of goblet cells and, by extension, GAPs (Fig. 3h–i).

### CD1d is expressed by colonic goblet cells and required for expansion of the colonic iNKT cell population

Stimulatory glycolipids the size of α-GalCer (~1kD) can cross the epithelial barrier by paracellular pathway^52,53^ causing us to question if there might be additional features to colonic GAP delivery of glycolipids required to stimulate colonic iNKT cells beyond acting as a conduit for accessing luminal substances. CD1d is expressed by a variety of cells including dendritic cells, B cells, innate lymphoid cells^11, 54, 55, 56, 57, 58^, and non-hematopoietic cells including intestinal epithelial cells^59^. CD1d expression by intestinal epithelial cells acts as a receptor stimulating IL-10 secretion to counterbalance inflammatory responses^34^. While the expression of CD1d on intestinal epithelial cells in general is well accepted, the studies evaluating CD1d expression by intestinal epithelial cells used approaches that deleted CD1d on all intestinal epithelial cell lineages and could not determine if CD1d expression and functions included, or was limited to, goblet cells.

**Fig. 4:**
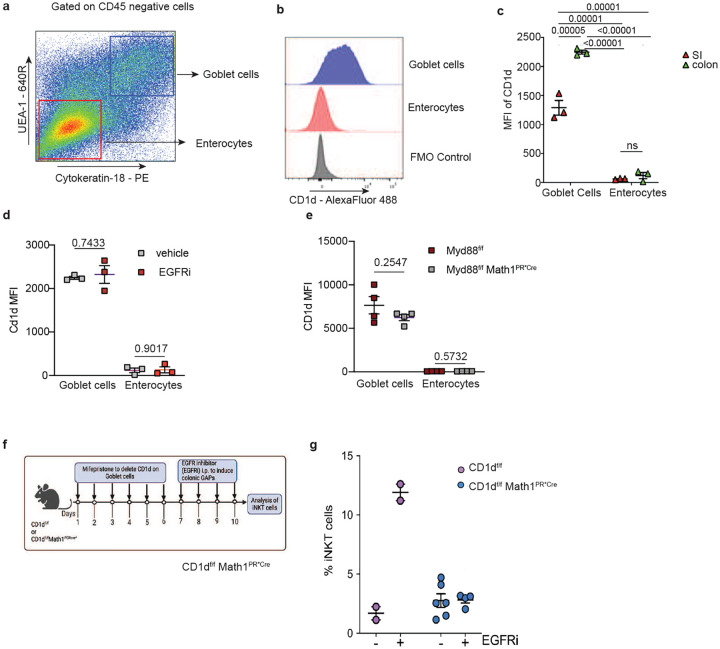
CD1d expression by goblet cells is required for expansion of colonic iNKT cells. **a** Representative FACS plot and **b** histogram of CD1d expression and graphs of Mean Fluorescence Intensity (MFI) of CD1d expression by goblet cells and enterocytes in the SI and colon at **c** steady state and following colonic GAP induction by **d** EGFRi treatment or **e** deletion of Myd88 in goblet cells. **f** Schematic of treatment to delete CD1d on goblet cells and open colonic GAPs and **g** frequency of iNKT cells in the colon LP of CD1d^f/f^ and CD1d^f/f^ Math1^PGRCre*^ mice (lacking CD1d in goblet cells) with or without EGFRi treatment. Each data point represents one mouse. Data presented here as mean SD and analyzed by a two-tailed student’s t test.

We observed that goblet cells (CD45^−^ UEA1^+^ Cytokeratin 18^+^ cells in the epithelial fraction) expressed CD1d at higher levels than intestinal enterocytes (CD45^−^ UEA-1^−^, Cytokeratin 18^−^ cells in the epithelial fraction) and that colonic goblet cells had higher CD1d expression than SI goblet cells (Fig. 4a–c). CD1d expression was not altered in colonic goblet cells by EGFR blockade or deletion of MyD88 in goblet cells (Fig. 4d and e), interventions that induced opening of colonic GAPs and expansion of colonic iNKT cells (above Fig. 3). To evaluate a functional role of CD1d in colonic goblet cells, we generated a mouse with inducible deletion of CD1d in goblet cells (CD1d^f/f^Math1^PR*Cre^ mice). Deletion of CD1d on goblet cells impaired expansion of colonic iNKT cells in response to EGFR blockade (Fig. 4f and g), supporting that CD1d expression by goblet cells has a functional role in the expansion of colonic iNKT cells when colonic GAPs were present. In addition, the requirement for CD1d expression by goblet cells to expand colonic iNKT cells suggests that to some extent goblet cells act in a non-traditional antigen presentation capacity to stimulate iNKT cells.

### Colonic GAPs preferentially expand iNKT cell populations with an intermediate transcriptomic signature

**Fig. 5:**
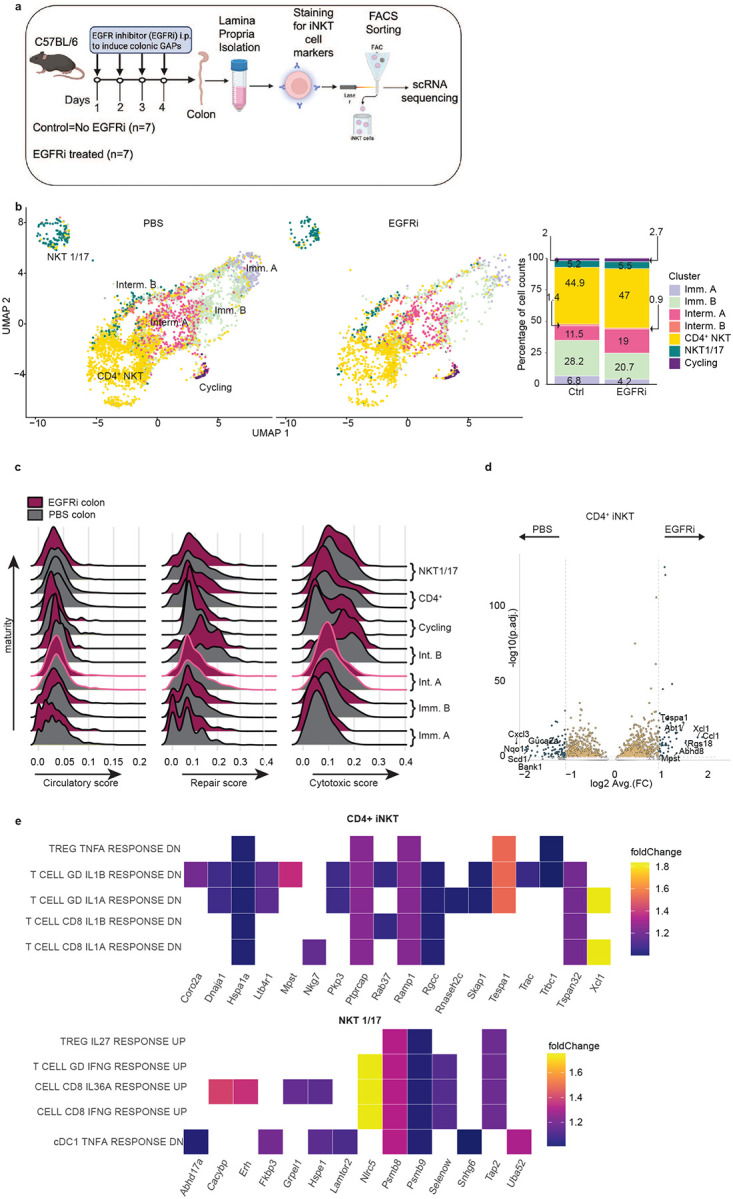
scRNA-seq of colonic iNKT cells from control and EGFRi treated mice reveals preferential expansion of the intermediate A population. **a** Schematic of scRNA-seq of sorted mouse colonic iNKT cells. **b** UMAP and bar plot of colonic iNKT cells from PBS or EGFRi treated mice demonstrates a preferential expansion of the intermediate A cluster and to a lesser extent the CD4+ and cycling populations. **c** EGFRi treatment did not induce a cytotoxic, repair, or circulatory signature, despite increased prevalence of CD4+ iNKTs. **d** Volcano plot of DEGs of CD4^+^ iNKT showed limited upregulation of genes. **e** Heatmap of upregulated genes from top 5 M7 MSigDB Gene Set enriched in EGFRi treated iNKTs from CD4^+^ and iNKT1/17 clusters. These genes are usually downregulated in T cells upon stimulation with inflammatory cytokines IL1α, IL1β, and TNFα and upregulated in response to IFNγ, IL36a, and IL27. Gene set abbreviation: DN: down, GD: T cell γδ.

The induction of colonic GAPs doubles the overall iNKT cell population. Given that the colonic iNKT cell population is heterogeneous containing immature, intermediate, cycling, and mature populations, we evaluated if expansion preferentially affected select iNKT cell populations over others. We performed scRNA-seq on sorted colonic iNKT cells following GAP induction (Fig. 5a schematic) and observed that there was a moderate increase in the Intermediate A cell population and more modest increase in the CD4^+^ iNKT cell and cycling populations largely at the expense of reducing the frequency of the Immature A & B cell populations (Fig. 5b). Because colonic GAP induction doubled the size of the iNKT cell population, all subsets were numerically expanded with the Intermediate A population being most affected.

**Extended Data Fig.3:**
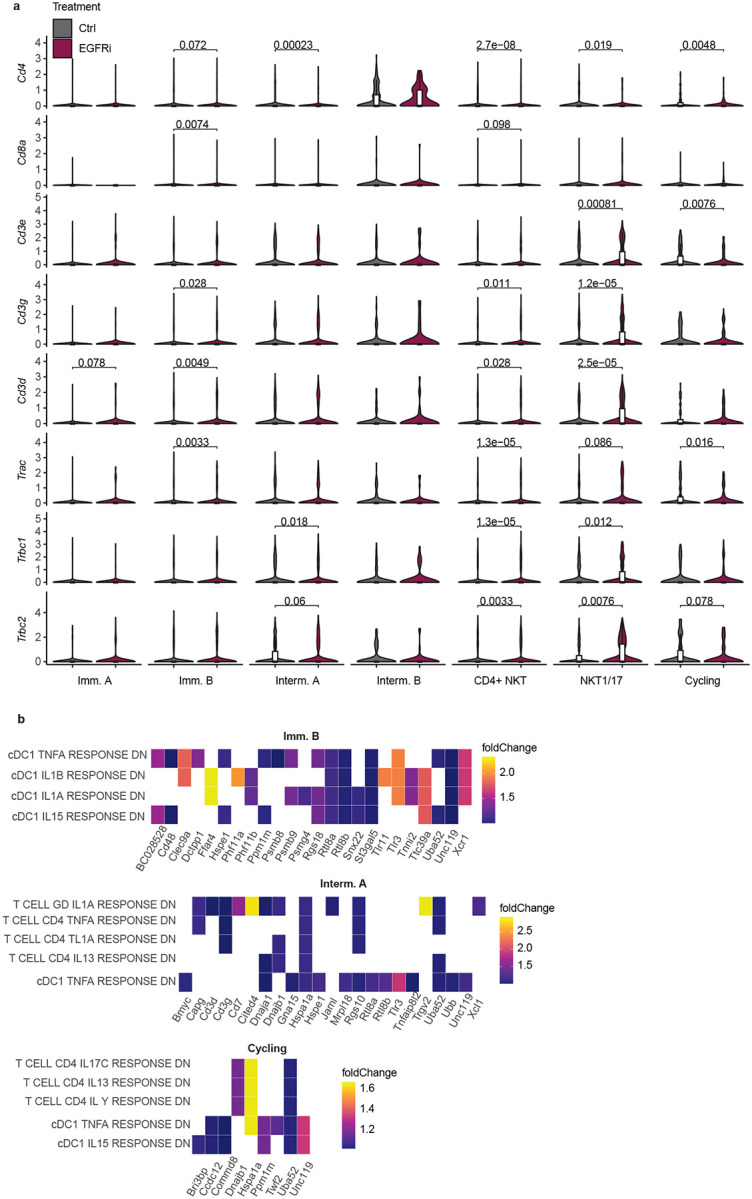
TCR activation signature in mature iNKT cell subsets upon EGFRi treatment. **a** Volcano plots of TCR components for each iNKT cluster showed upregulation mainly in the mature subsets following opening of colonic GAPs with EGFRi treatment. **b** Heatmap of top 5 M7 MSigDB Gene Sets upregulated in EGFRi treated colonic iNKTs. These genes are usually downregulated in T cells upon stimulation with inflammatory cytokines IL1α, IL1β, IL13, IL15, IL17c, and TNFα. Gene set abbreviation: DN: down, GD: T cell γδ.

Of note the previously reported cytotoxic, repair, or circulatory gene signatures were not upregulated upon colonic GAP opening (Fig. 5c). However, the terminally differentiated CD4^+^ iNKT population showed upregulated genes associated with T cell signaling and activation following GAP induction (Fig. 5d, Extended Fig. 3a and Extended Data Table 2), consistent with colonic GAPs delivering stimulatory glycolipids. Within the top upregulated genes were two chemokines *Xcl1* and *Ccl1. Xcl1* produced by iNKTs has been associated with recruiting CD103^+^ DCs in the airways^60^ while *Ccl1* has generally been thought to be produced mainly by conventional CD4^+^ and CD8^+^ T cells and macrophages in the gut^61^. Notably, *Ccl1* has been linked to improved outcomes in DSS colitis models by inducing IFNγ expression in ILCs^61^. Among the downregulated genes, *Cxcl3* stood out, as it has not been reported to be produced by iNKTs before. Given this chemokine is important for the recruitment of neutrophils, its downregulation could contribute to a less inflammatory environment.

Analysis revealed only a few genes were upregulated by EGFR inhibition in other clusters which did not translate to enrichment in defined pathways. When compared to the Immunological Gene Signature Set M7 (GSEA^62^) the top 5 gene sets enriched in CD4^+^ iNKT cells in response to EGFRi were T cell signatures downregulated in response to inflammatory cytokines (IL1α, ILβ, TNFα). In contrast EGFRi induced genes in the NKT 1/17 cluster which are upregulated by IFNγ, IL27 and IL36A (Fig. 5e). Similar to CD4^+^ iNKT cells, the Immature B, Intermediate A and cycling iNKT cell clusters upregulated gene signatures that are downregulated by inflammatory cytokines in T cells (Extended Figure 3b). In summary, colonic GAPs induced activation in mature iNKT cell populations however this resulted in induction of genes that are downregulated in presence of inflammatory cytokines, suggesting that induction of colonic GAPs shifts the iNKT cell population towards a less inflammatory phenotype.

### Colonic iNKT cell expansion is durable and associated with improved outcomes in a colitis model

**Fig. 6:**
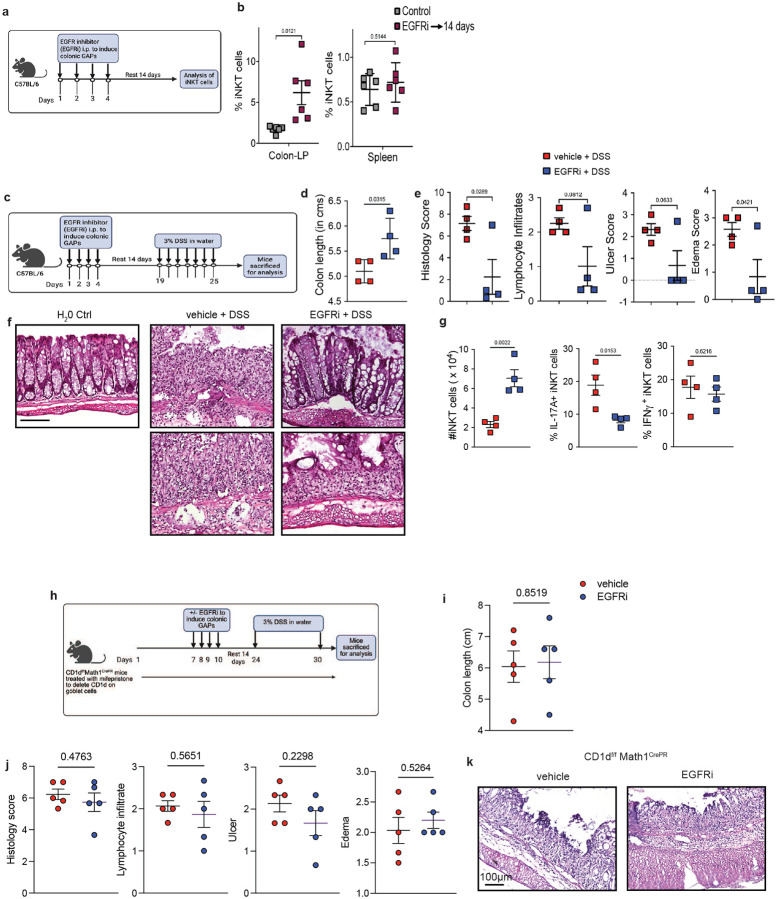
Colonic iNKT expansion is durable and protective in DSS-Colitis. **a** Schematic of model to evaluate persistence and **b** frequency of colonic and splenic iNKT cells 14 days following colonic GAP induction. **c** Schematic of DSS-colitis model following iNKT cell expansion. **d** Colon length, **e** overall histology score and individual components scores, **f** numbers of total colonic iNKT cells and IL17 and IFNγ expression by colonic iNKT cells, and **g** representative photomicrographs of the colon of mice receiving vehicle or EGFRi 14 days prior to DSS. **h** Schematic of CD1d deletion on goblet cells, treatment to induce colonic GAPs, and induction of DSS colitis. **i** colon length, **j** total histology scores and individual component scores and **k** representative photomicrographs of the colon of mice lacking CD1d on goblet cells and receiving vehicle or EGFRi 14 days prior to DSS. Each data point represents and individual mouse. Data is presented as the mean +/− SEM. Panels i-k representative of one of two experiments. Each data point represents one mouse. Data represented here is mean SD and analyzed by a two-tailed student’s t test.

Colonic iNKT cells have been viewed as a static, tissue-resident population that established in early life. Consistent with this concept, we did not see a strong circulatory signature in colonic iNKT cells from humans or mice at baseline (Fig. 1e). We evaluated the durability of the colonic iNKT cells two weeks following expansion by EGFRi and observed that the population remained unchanged (Fig. 6a–b). Further, consistent with this tissue resident phenotype and lack of migration, we did not observe changes in the splenic iNKT cell population (Fig. 6b).

iNKT cells have been observed to impact outcomes in mouse colitis models with studies demonstrating both beneficial and detrimental effects of iNKT cells^6, 31, 32, 33, 63, 64, 65, 66, 67^. Therefore, we evaluated the effect of expansion of the colonic iNKT population on intestinal inflammation using the dextran sodium sulfate (DSS) colitis model. Colonic iNKT cells were expanded by EGFRi treatment, mice were rested for two weeks to allow for washout of any residual drug, then given 3% DSS in drinking water (Fig. 6c). Colons from mice treated with EGFRi had less colonic shortening, improved histology scores (reduced edema, ulcers, and lymphocyte infiltrates; Fig. 6d–f). Further, we observed that the expansion of colonic iNKT cells persisted during DSS colitis, and that while the percentage of IFNγ producing iNKT cells was unchanged, there were significantly fewer iNKT cells expressing IL-17 (Fig. 6g).

Because EGFRi might also have effects independent of expansion of colonic iNKT cells that could impact colitis, we evaluated the effects of EGFRi treatment in mice with CD1d deficient goblet cells, where we did not observe colonic iNKT cell expansion after EGFRi treatment (Schematic Fig. 6h and data Fig. 4g). Deletion of CD1d on goblet cells reversed the protective effects of EGFRi treatment on DSS colitis, including colon shortening and histology scores (Fig. i-k), consistent with the protective effect of EGFRi being mediated through CD1d expression by goblet cells and colonic iNKT cell expansion. Together, these results demonstrate that colonic iNKT cell expansion via colonic GAPs is durable, tissue restricted and can be protective in colitis in some settings.

## Discussion

iNKT cells express a semi-invariant T cell receptor that recognizes glycolipid antigens presented on the non-polymorphic MHC I like molecule CD1d and combine features of both innate and adaptive immunity^12^. iNKT cells and CD1d are evolutionarily conserved across mammals, with invariant T cell counterparts and CD1d like molecules also being found in lower species including amphibians^68,12^. iNKT cells are enriched at barrier surfaces and perform critical roles in immunity as early responders recruiting other immune cells and directing the tone of the ensuing outcomes. iNKT cells and mucosa associated invariant T (MAIT) cells share unconventional processes for selection/development in the thymus that is initiated when double positive thymocytes expressing the semi-invariant iNKT T cell receptor or MAIT T cell receptor interacts with double positive thymocytes expressing CD1d or the MHCI related protein MR1, respectively. This results in homotypic interactions of signaling lymphocytic activation molecule (SLAM) family receptors between the double positive thymocytes and starts the signaling/developmental pathway towards differentiated iNKT or MAIT cell populations. Historically, iNKT cells were thought to complete their development/differentiation in the thymus and subsequently seed peripheral sites as fully developed, or committed, cellular populations. However, emerging studies suggest that peripheral iNKT cells can retain plasticity and may exist in intermediate stages of development in some settings^42, 69, 70^.

Most studies of iNKT cell subsets have focused upon the thymus with fewer comparing peripheral iNKT cells to their thymic progenitor/counterparts and no studies evaluating the colonic iNKT cell populations at the single cell resolution. Here, we find that human and mouse colonic iNKT cells share similar transcriptomic profiles to the thymic iNKT cell populations including the presence of populations with immature, intermediate, and cycling transcriptomic signatures. Moreover, trajectory analysis revealed a parallel pathway of predicted development in colonic and thymic iNKT cell populations, further suggesting that colonic iNKT cells are not fully differentiated or fixed in identity. The flexibility of the colonic iNKT cell population might be further amplified by the variety of stimulatory or modulatory self and environmental/microbial glycolipid antigens and the variety of CD1d expressing cellular populations present within the gut, offering additional approaches to modulate gut iNKT cell responses.

These findings contrast with the view that the colonic iNKT cell population once established in early life is ‘fixed’ in size and phenotype. Supporting this concept, germ free mice have an expanded colonic iNKT cell population when compared with specific pathogen free (SPF) housed mice, and recolonization of germ free mice with SPF gut microbiota prior to weaning, but not after, limits the expansion of the colonic iNKT cell population to SPF housed mouse levels^31^. Further, supporting a time-limited role for the gut microbiota constraining the size of the colonic iNKT cell population in early life, the developed gut microbiota suppresses embryonic macrophages that play a role in facilitating iNKT cell residency in the colon^36^ and some microbes present in the mature gut microbiota produce sphingolipids, which act as immunomodulatory glycolipid antigens suppressing iNKT cells^35^. Early life manipulations in the colonic iNKT cell population are durable and can confer long-term predisposition to disease outcomes^36^. Consistent with this we observed that the SI, but not the colonic, iNKT cell population could be expanded by administering exogenous luminal stimulatory glycolipid antigens.

However, we observed that the inability to expand the colonic iNKT cell population in response to luminal stimulatory glycolipid antigens was not a property of the colonic iNKT cells or their environment *per se*, but rather due to inability of luminal stimulatory glycolipid antigens to be encountered by, and presented to, colonic iNKT cells. Opening of colonic GAPs, which are normally suppressed by the abundant microbiota post-weaning, was sufficient to expand the colonic iNKT cell population. This demonstrates that stimulatory glycolipid antigens are present in the colonic lumen and that they are delivered via GAPs. While we don’t know the physical properties of these glycolipids, molecules the size of α-GalCer (~1kD), should be able to traverse the epithelium via the paracellular leak pathway^53^, raising the possibility that colonic GAPs may be doing more than allowing stimulatory glycolipids to access the lamina propria immune compartment. Indeed, we found that deletion of CD1d on goblet cells abrogated colonic iNKT cell expansion when colonic GAPs were induced. Consistent with the concept of CD1d on colonic goblet cells acting in an antigen presentation capacity, prior studies found systemically administered fluorescent α-GalCer localized to the colonic epithelium of wildtype but not CD1d deficient mice^67^. Furthermore, scRNA-seq analysis revealed increased expression of genes related to T cell activation when colonic GAPs were induced, consistent with iNKT cells being stimulated by their cognate antigen presented by CD1d. These observations indicate that colonic goblet cells act as unconventional antigen presenting cells and/or may pass off glycolipid antigens and CD1d to antigen presenting cells in the lamina propria, as we have observed lamina propria antigen presenting cells can acquire goblet cell proteins when they sample from GAPs^47^.

Maneuvers that induce GAPs in the colon did not expand the SI iNKT cell population. We interpret this to be due to the lack of increase in SI GAPs above baseline by these by these maneuvers^49, 71^. However, it may also reflect that glycolipid antigens more easily traverse the small intestinal epithelium via the paracellular pathway due to the thinner and more penetrable mucus^72^. Relatedly, we observed that the small intestinal goblet cells express lower levels of CD1d, potentially consistent with them performing a different role than colonic goblet cells in iNKT cell biology.

There is a growing understanding of the diversity of iNKT cells, including differentiated subsets that parallel T helper cell and innate lymphoid cell subsets, thymic populations that are less differentiated, and peripheral populations which may have some degree of plasticity^20, 30, 42, 73, 74^.^20, 30, 42, 73, 74^. In addition, there is a standing and growing appreciation that the iNKT cell populations in the periphery can vary by tissue^74^. In total these observations refine an older concept that all iNKT cells leave the thymus in differentiated or committed states and suggest that peripheral iNKT cells might be manipulated not to just change the size, but also the phenotype, of the iNKT cell population, and alter immune outcomes. Colonic GAP induction doubled the size of the colonic iNKT cell population. By scRNA-seq analysis expansion preferentially affected the intermediate A population, however given the relatively small compensatory changes in the proportions of the other colonic iNKT cell populations, all populations increased to some degree. The expansion of the colonic iNKT cell population was accompanied by expression of a reduced inflammatory transcriptomic signature, suggesting that the expanded iNKT cells may be less pro-inflammatory. Indeed, we observed that at baseline the colonic iNKT cell population more favored a repair signature when compared with iNKT cells in the thymus. Consistent with this, we observed that induction of colonic GAPs, which expanded the colonic iNKT cell population, improved outcomes in the DSS colitis model. The improved outcome is at least in part attributable to iNKT cells, as deletion of CD1d on goblet cells, which inhibited colonic iNKT cell expansion in response to GAP induction, abrogated the protective effect of GAP induction on DSS colitis. This is consistent with earlier studies demonstrating that systemic α-GalCer improved outcomes in DSS colitis in wildtype mice, but not in global CD1d deficient mice^67^. In addition, it is likely that the expanded colonic iNKT cell population recruits in additional immune cell populations prior to colitis induction which may have beneficial effects in this setting, as scRNA-seq analysis revealed increased expression of genes for chemotactic proteins.

In conventionally housed, unmanipulated mice colonic GAPs are physiologically present during a restricted period in early life initiated by decreasing concentrations of EGF in breastmilk and terminated by the establishment of the fully developed gut microbiota^49, 75^. During this period colonic GAPs play beneficial roles in promoting tolerance to dietary and microbial antigen, shaping the intestinal immune system, and facilitating the physiologic and beneficial translocation of some gut resident bacteria^49, 75, 76^. However, beyond this period in early life colonic GAPs can be experimentally induced by manipulations altering the gut microbiota and/or EGFR signaling^2, 51, 71^. Prior studies have suggested that opening of colonic GAPs later in life due to these manipulations has detrimental effects including promoting inflammation and facilitating the translocation and dissemination of enteric pathogens^71, 77^. In contrast, our finding here suggest that opening colonic GAPs postweaning can have beneficial outcomes in some contexts, raising the question if there are everyday situations in which colonic GAPs may be open in adults. Interestingly even a single clinically relevant dose of some antibiotics or exposure to subtherapeutic levels of antibiotics that might be encountered in contaminated food or water, can induce sufficient gut microbial dysbiosis to allow colonic GAPs to open^71, 78^. Thus, the presence of colonic GAPs beyond this period in early life, while not entirely physiologic, may be more common and playing a role in intestinal immunity than previously appreciated.

iNKT cells are a small population of potent cells rapidly responding to a restricted set of self and environmental glycolipids. These potent responders direct subsequent immune outcomes making them attractive targets for immunotherapy. While iNKT cell diversity within peripheral tissues is increasingly appreciated, the extent of this diversity and their plasticity remain to be defined. Our findings identify colonic iNKT cells as a dynamic, flexible population that can be expanded in adulthood through goblet cell–dependent antigen delivery. This expanded colonic iNKT cell population conferred protection in a colitis model, highlighting the therapeutic potential of peripheral iNKT cell manipulation. Moreover, our work uncovers an unanticipated role for intestinal goblet cells as nontraditional antigen presenting cells that shape intestinal immunity through CD1d-mediated glycolipid presentation. Together these findings highlight the flexibility and potential of colonic iNKT cells as effectors in mucosal immunity and position goblet cells as regulators of this axis.

## Materials and Methods

### Humans

All human studies procedures and protocols were approved by the institutional review board at Washington University in Saint Louis School of Medicine. Informed consent was obtained from patients, ranging from 20 to 73 years of age, and resection specimens (approximately 1 by 2 inches) were collected during surgeries performed at Barnes Jewish Hospital by the Digestive Diseases Research Core Centers Biobank at Washington University School of Medicine in St. Louis, Missouri, United States.

### Mice

All mice used in these studies were on the C57BL/6 background. Mice were 8–16 weeks of age at the time of analysis. Mice were housed in a 12-hour light dark cycle in a temperature-controlled room in a specific pathogen free facility, fed a routine chow diet, and bred in house at Washington University in Saint Louis School of Medicine, St. Louis, Missouri, United States. EGFR^f/f^ mice^79^ were a gift from Dr. David Threadgill, (University of North Carolina). CD11c-YFP^80^ were a gift from M. Nussenzweig (The Rockefeller University, New York, New York). Math1^f/f^ mice^81^, MyD88^f/f^ mice^81^ and Math1^PR*Cre^ mice^82^ were purchased from The Jackson Laboratory (Bar Harbor, ME). CD1d^f/f^ mice were generated previously^34^. MyD88^f/f^ mice, EGFR^f/f^ mice, and CD1d^f/f^ mice, were bred to Math1^Cre*PR^ mice to generate an inducible deletion of MyD88, EGFR and CD1d in goblet cells, respectively. Transgenic mice with tamoxifen-dependent Cre recombinase are expressed under the control of villin promoter (vil-Cre-ERT2 mice^83^) and were a kind gift from Sylvie Robine (Institut Curie, Paris, France). Math1^f/f^ mice were bred to vil-Cre-ERT2 mice to generate mice with inducible depletion of goblet cells following deletion of Math1 in villin expressing cells. Math1^PR*Cre^ mice were injected intraperitoneally (i.p.) with RU486 (mifepristone) (10mg/kg) dissolved in sunflower seed oil or given RU486 (250mg/L) in drinking water daily for 6 days. Math1^f/f^vil-Cre-ERT2 mice were injected with tamoxifen as previously described^2^. Mice of both sexes and Cre negative littermate controls were used for experiments. 2μg α-galactosyl ceramide was given by gavage or enema using a 16G plastic cannula inserted 3 cm transanally into the colon and analyzed 24 hours later. 6.6μg Tryphostin AG1478 (Sigma Aldrich) was injected i.p. or vehicle for 4 days prior to analysis. Animal procedures and protocols were carried out in accordance with the institutional review board at Washington University School of Medicine in St. Louis.

### Isolation of murine lamina propria and Intestinal epithelial cells:

Epithelial cells and lamina propria mononuclear cells were isolated as previously described^84^. The epithelial fractions/supernatants of first wash were used for staining intestinal epithelial cells and CD1d staining. Epithelial cells and lamina propria mononuclear cells were isolated as previously described^84^. The epithelial fractions/supernatants of first wash were used for staining intestinal epithelial cells and CD1d staining.

### Isolation of Spleen:

Spleen were minced using plunger on 70-μm cell strainers and washed with PBS, centrifuged for 5 mins at 1,500 rpm and resuspended in PBS before performing the cell staining with antibodies for flow cytometry.

### Isolation of human colonic lamina propria and Intestinal epithelial cells:

Colonic resections were stored in MACS storage solution prior to processing. For lamina propria isolation, white fatty muscularis layer was removed and tissues washed with 40ml Hank’s Balanced Salt solution (HBSS) containing 5mM of EDTA for 30 mins for 2 times at37°C in a shaking incubator. The epithelial fractions of each wash were pooled and used for analysis of CD1d expression by flow cytometry. Remaining tissues were cut into pieces and digested with Dispase (50U/ml stock) and Type VIII Collagenase (10,000 U/ml) as above for murine lamina propria mononuclear cell isolation at 37°C under shaking conditions for 90 mins in RPMI +5% FBS media. The digested tissues were filtered through 100-μm cell strainer and centrifuged at 1,500 rpm for 5 mins. The cells were then resuspended in PBS before performing the cell staining for flow cytometry staining.

### Flow Cytometry

Lamina propria cellular populations were incubated with mouse or human loaded CD1d (PBS-57) or unloaded tetramers (NIH Tetramer Core Facility at Emory) for 45 mins diluted in (PBS + 1% BSA + Human Ig 1mg/ml) on ice. Following incubation, cells were washed stained for cell surface proteins for 20 mins on ice. For intracellular cytokine staining, cells were incubated with cell stimulation cocktail at 37°C for 45 mins then washed, stained for cell surface markers as above, fixed and permeabilized and stained for intracellular markers per the manufacturer’s recommendations. For CD1d surface staining, epithelial cells were incubated for 20 mins on ice with primary antibodies. Data acquisition and analysis was performed using Attune NXT software and analyzed using FlowJo software. Reagents are listed in supplementary Table 2.

### Single cell scRNA-seq Sequencing

Mouse colonic-LP cells were isolated as above from Control (n=7) and EGFRi treated mice (n=7) and sorted as live (viability dye) iNKT Cells (CD45^+^CD3^+^TCRβ^+^PBS-57-loaded CD1d tetramer^+^) using an BD FACS Aria Fusion cell sorter (Purity >95%) into RPMI containing 10% FCS. Sorted cells were counted and 10,000 cells were loaded onto a 10X Genomics Chromium controller for 3’v3.1 library preparation as per the manufacturer’s recommendations. The concentration of each library was determined through qPCR utilizing the KAPA library Quantification Kit according to the manufacturer’s protocol (KAPA Biosystems/Roche) to produce cluster counts appropriate for the Illumina NovaSeq6000 instrument. Normalized libraries were sequenced on a NovaSeq6000 S4 Flow Cell using the XP workflow and a 50x10x16x150 sequencing recipe according to manufacturer protocol. A median sequencing depth of 50,000 reads/cell was targeted for each Gene Expression Library. Library preparation and sequencing was performed at the Genome Technology Access Center at Washington University in Saint Louis School of Medicine.

Human colonic-LP cells were isolated as described above and sorted for live (Ghostdye 780) iNKT Cells (CD45^+^CD3^+^TCRβ^+^PBS-57-loaded CD1d tetramer^+^) using a BD FACS Aria Fusion cell sorter (Purity > 95%) into Pipseq Cell Suspension Buffer with RNAseOUT. Sorted iNKT cells were processed with the T2 PIPseq kit following manufacturer instructions prior to sequencing on Illumina NovaSeq6000 S4 Flow Cell using the XP workflow and a 50x10x16x150 sequencing recipe according to manufacturer protocol at the Genome Technology Access Center at Washington University School of Medicine in St. Louis.

### Single cell RNA-seq analysis

Fastq files from the human data were further processed with the PIPseeker V 2.1.4 to obtain UMI tables for each sample. Fastq files for the mouse data were processed with 10x Genomics Cell Ranger to obtain UMI tables for each condition. The resulting UMI tables were processed in *R* with the Seurat^85^ package to perform quality control, integration, annotation, and differential abundance analysis. Contaminating non-immune cells were removed by filtering based on CD45 gene expression (P*trcp)*. The high prevalence of plasma cells in the colon and their fragility during digestion and isolation can result in release of immunoglobulin transcripts. Immunogolobulin genes were removed as transcriptional contaminants; we did not observe cellular populations with gene expression profiles consistent with plasma cells or B cells. We used the previously published thymic iNKT cells from Bugaut e*t al*., to perform anchoring of our colonic human iNKT and mouse after determining orthologs with the orthologous matrix^86^. The thymic iNKT cells were normalized and filtered according to the metrics provided by Bugaut et *al. t*o recreate the UMAP clustering for annotation. After annotation the human and mouse colonic data ware anchored to the thymic iNKTs and predicted annotations were transferred. For the human data we performed differential gene expression analysis based on sex. For the mouse data we performed differential gene expression based on treatment condition for individual clusters with FindMarkers. Pathway enrichment was determined with the enricherGO function of clusterProfiler^87^ (and enricher function against the mouse MSigDB M7 for immunological gene signature sets. Prior to differential gene expression ribosomal and mitochondrial genes were filtered out. For trajectory analysis we used the slingshot^88^ package to determine pseudotime. Scores for different gene profiles were calculated in Seurat with AddModuleScore function. Volcano plots were generated with ScPubr^89^, heatmaps of enriched pathways with clusterProfiler heatmap function and UMAP and feature plots with SeuratExtend^90^.

### Intravital two-photon microscopy

In vivo two photon imaging was performed as previously described^47^. Mice were anesthetized with nebulized isoflurane in in 95% O2/5% CO2. Intravital microscopy preparation of small intestinal loops were performed as follows. A small incision was made through the skin and peritoneum exposing the small intestine. To image the lumen, an incision was made and *in vivo* two photon imaging was performed as previously described^47^. Mice were anesthetized with nebulized isoflurane in 95% O2/5% CO2. Intravital microscopy preparation of small intestinal loops were performed as follows. Fluorescently labeled dextran 10,000MW (10mg/ml, Invitrogen, Carlsbad, CA) and dansylated α-galactosyl ceramide (1mg/ml) were injected into the lumen.

### DSS Colitis model

C57BL/6 mice were treated with EGFRi and CD1d^f/f^Math1^PGRCre*^ mice were treated with RU486 or vehicle and EGFRi as outlined in the schematics, rested for 14 days, then given 3%(w/v) dextran sodium sulfate (DSS; molecular weight 36–50kDa) in autoclaved drinking water for 7 days. Weight, stool pellets and rectal bleeding were monitored daily. At day 8, distal colonic tissue specimens were processed for analysis.

### Statistical Analysis

Single cell RNA sequencing data was analyzed as above. Data was analyzed using GraphPad Prism (GraphPad Software Inc., San Diego, CA). A standard two-tailed Student’s t-test or one-way analysis of variance (ANOVA) with Tukey or Dunnett test was used to determine the statistical significance (unless stated otherwise).

## Extended Data

**Extended Data Table 1:** 

Identifier	Age	Sex	Race	Case	anti-inflammatory	steroids	Other Medical Conditions
LIB040313	20	M	White	XI Ileocecectomy	Ibuprofen, Montelukast		Asthma, Crohn’s
LIB040314	70	F	White	Sigmoid Resection for Diverticulitis	acetaminophen		
LIB040315	63	M	White	Sigmoid Resection for Diverticulitis		hydrocortisone	
LIB040316	52	F	Black	Ileocolic Resection for neuroendocrine carcinoma	acetaminophen		Morbid obesity; gastroesophageal reflux disease
LIB040317	41	M	White	Left Colon Resection for Diverticulitis	acetaminophen, ibuprofen		
LIB040318	51	F	White	Right Colon Resection for Colon Polyp			small bowel resection, Chronic allergic conjunctivitis
LIB040319	45	M	White	Left Colon Resection for colon polyp	acetaminophen, ibuprofen		Clostridium difficile infection

## Supplementary Material

1

## Data Availability

Human and mouse single cell RNA sequencing data are available via Gene Expression Omibus GSE306154. To review GEO accession GSE306154: Go to https://nam10.safelinks.protection.outlook.com/?url=https%3A%2F%2Fwww.ncbi.nlm.nih.gov%2Fgeo%2Fquery%2Facc.cgi%3Facc%3DGSE306154&data=05%7C02%7Crnewberry%40wustl.edu%7C6f6a0f010c5e48029f9908ddf6e8d91b%7C4ccca3b571cd4e6d974b4d9beb96c6d6%7C0%7C0%7C638938202114273312%7CUnknown%7CTWFpbGZsb3d8eyJFbXB0eU1hcGkiOnRydWUsIlYiOiIwLjAuMDAwMCIsIlAiOiJXaW4zMiIsIkFOIjoiTWFpbCIsIldUIjoyfQ%3D%3D%7C0%7C%7C%7C&sdata=2rdMPlaHkxEhFZkYxNEhyotGdwqTwzgYqTjyiKfJnag%3D&reserved=0Enter token ovqhkuqudduldgx into the box.
