# Supplementary material for: CD1d+ Goblet Cells Expand Colon-Resident Immature, Intermediate and Differentiated iNKT Cells to Limit Colitis
Source: Res Sq. 2025 Sep 25:rs.3.rs-7603392. Preprint. [Version 1] doi: 10.21203/rs.3.rs-7603392/v1 (PMC12486123; doi:10.21203/rs.3.rs-7603392/v1)
Supplement: 1 [file NIHPPRS7603392V1-supplement-1.pdf]

Supplementary Table 2

REAGENT or RESOURCE	SOURCE	IDENTIFIER
**Antibodies**		
anti-Human CD45-eFlour 450 (Clone 2D1)	ebioscience	cat # 48-9459-42 RRID:AB_1603240
anti-Human CD3-APC (Clone OKT3)	ebioscience	cat # 17-0037-42 RRID:AB_1907373
anti-Human TCRβ-PE Cy7 (Clone IP26)	ebioscience	Cat#12-9986-42 RRID:AB_2573572
anti-human CD1d PBS-57-tetramer-PE	NIH tetramer facility	
anti-human CD1d PBS-57-tetramer-APC	NIH tetramer facility	
anti-mouse CD1d PBS-57 tetramer-PE	NIH tetramer facility	
anti-mouse CD1d PBS-57 tetramer-APC	NIH tetramer facility	
anti-mouse CD45-PE Cy7 (Clone: 30-F11)	Thermo Fisher Scientific	Cat# 25-0451-82, RRID: AB_2734986
anti-mouse CD45-eFlour506 (Clone: 30-F11)	Thermo Fisher Scientific	Cat# 69-0451-80, RRID: AB_2637146
anti-mouse CD3e-eFlour450 (Clone:145-2C11)	Thermo Fisher Scientific	Cat# 48-0031-82, RRID: AB_10735092
anti-mouse CD3e-APC (Clone:145-2C11)	Thermo Fisher Scientific	Cat# 17-0031-83, RRID: AB_469316
anti-mouse TCRβ-APC-eFlour780 (Clone:H57-597)	Thermo Fisher Scientific	Cat# 47-5961-82, RRID: AB_1272173
anti-mouse TCRβ-FITC (Clone:H57-597)	Thermo Fisher Scientific	Cat# 11-5961-82, RRID: AB_465323
UEA-1-640R	Biotinum	Cat# 29112
UEA1 Fluorescein	Vector laboratories	Cat# FL-1061
anti-CD69-PerCP Cyanine 5.5 (Clone: H1.2F3)	Thermo Fisher Scientific	Cat# 45-0691-82, RRID: AB_1210703)
anti-mouse CD1d-Alexa 488 (Clone: 1B1)	Thermo Fisher Scientific	Cat# 53-0011-82, RRID: AB_1944357

Cytokeratin-18 Biotin	Abcam	Cat# ab668, RRID: AB_305647)
anti-mouse CD44-eFlour 450<br>(Clone:IM7)	Thermo Fisher Scientific	(Cat# 48-0441-82, RRID: AB_1272246
anti-mouse CD62L-Super Bright 600<br>(Clone: MEL-14)	Thermo Fisher Scientific	Cat# 63-0621-80, RRID: AB_2637415)
anti-mouse Ifnγ-Alexa 488<br>(Clone: XMG1.2)	Thermo Fisher Scientific	Cat# 53-7311-82, RRID: AB_469932
anti-mouse Il17α-APC<br>(Clone: eBio17B7)	Thermo Fisher Scientific	Cat# 17-7177-81, RRID: AB_763580
Streptavidin-APC	Molecular Probes	SA1005
Streptavidin-PE	Molecular Probes	SA10041
Streptavidin-PE Cy7	Thermo Fisher Scientific	Cat# 25-4317-82, RRID: AB_10116480
anti mouse-CD45.1-PE-Cy7<br>(Clone: A20)	Thermo Fisher Scientific	Cat# 25-0453-81, RRID: AB_469628)
anti mouse-CD45.2-FITC<br>(Clone:104)	Thermo Fisher Scientific	Cat# 11-0454-81, RRID:AB_465060)
Live/Dead Fixable violet Dead cell stain kit	Invitrogen	Cat # L34964
